# Associations between corticosteroid dosage and clinical outcomes in patients with hypoxemic COVID-19 pneumonia: A retrospective cohort study

**DOI:** 10.1371/journal.pone.0308069

**Published:** 2024-09-06

**Authors:** Napassorn Teeratakulpisarn, Supattra Chiewroongroj, Thummaporn Naorungroj, Ranistha Ratanarat

**Affiliations:** Department of Medicine, Faculty of Medicine Siriraj Hospital, Mahidol University, Bangkok, Thailand; University of Toronto, CANADA

## Abstract

**Background:**

Corticosteroids are commonly used to treat COVID-19 patients with hypoxemia, and clinicians have adjusted the corticosteroid intensity on the basis of clinical needs. However, neither the optimal dose nor the duration of treatment has been recommended.

**Objective:**

To investigate whether cumulative doses of corticosteroids, measured as dexamethasone-equivalent doses over the first 14 days, impact outcomes in patients with COVID-19 pneumonia.

**Methods:**

We conducted a retrospective cohort study of COVID-19 pneumonia patients admitted between April 1^st^, 2020, and September 30^th^, 2021. The study focused on the type and dose of corticosteroid administered during the initial 14 days, clinical outcomes, and complications. The primary outcome was in-hospital mortality.

**Results:**

Among 271 patients, the mean cumulative dexamethasone-equivalent dose was 158 (119.9–197.25) mg in survivors and 185 (131.7–222.0) mg in nonsurvivors. Univariate analysis revealed that the cumulative dexamethasone-equivalent dose was a risk factor for in-hospital mortality. However, this association did not hold true in the multivariate analysis. After the cumulative dexamethasone-equivalent dose was categorized into quartiles, the moderate dosage (126.01–165.00 mg) in the second quartile was found to be associated with the lowest in-hospital mortality (16.2%). Higher cumulative dexamethasone-equivalent doses were associated with longer hospital and ICU stays and fewer ventilator-free days (p < 0.001). Doses exceeding 165 mg were associated with an increased risk of hospital-acquired infections (p < 0.001).

**Conclusions:**

The cumulative dexamethasone-equivalent dose during the first 14 days is not associated with in-hospital mortality in hypoxemic COVID-19 patients. However, higher cumulative doses exceeding 165 mg are associated with an increased risk of in-hospital mortality and secondary hospital-acquired infections.

## Introduction

COVID-19 is an infectious disease caused by severe acute respiratory syndrome coronavirus 2 (SARS-CoV-2), which emerged in late 2019 and was declared a pandemic by the World Health Organization (WHO) in March 2020 [1]. The severity of the disease ranges from asymptomatic to severe pneumonia with acute respiratory distress syndrome and fatal outcomes, particularly in patients with comorbidities, who have a greater potential risk of severe COVID-19 [2]. The pathogenesis of COVID-19 involves two main processes: an early phase driven by SARS-CoV-2 replication and a later phase characterized by immune dysregulation and an inflammatory response, leading to tissue damage. Early-phase treatment primarily involves antiviral therapy, whereas immunosuppressive and anti-inflammatory therapies are more beneficial in the later stage [3].

Immunomodulatory therapy for COVID-19 mainly relies on corticosteroids, particularly dexamethasone. However, dexamethasone has inconsistent outcomes depending on oxygen dependence. Landmark studies, such as the Randomized Evaluation of COVID-19 Therapy (RECOVERY) trial and the COVID-19 Dexamethasone (CODEX) trial, demonstrated that the use of dexamethasone showed benefits of mitigating disease severity and death in COVID-19 patients receiving oxygen therapy [4, 5]. Conversely, a systematic review and meta-analysis revealed that the use of dexamethasone in patients not requiring oxygen was linked to increased disease severity and mortality risk [6]. Moreover, studies using alternative corticosteroids (e.g., methylprednisolone and hydrocortisone) in COVID-19 patients reported no improvement in clinical outcomes or mortality rates [7–10]. Consequently, the current National Institute’s COVID-19 treatment guidelines recommend the use of dexamethasone only in patients requiring oxygen therapy [3]. Although one study did compare high- vs. low-dose corticosteroids, it did not report statistically significant outcomes [11]. A consensus on the optimal dose and duration of dexamethasone treatment is still needed.

Therefore, we investigated whether corticosteroid intensity, measured by the cumulative dexamethasone-equivalent dose over the first 14 days, affects in-hospital mortality in patients with COVID-19 pneumonia.

## Materials and methods

### Study design

This single-center retrospective observational study was conducted at Siriraj Hospital, a tertiary hospital affiliated with Mahidol University, Thailand, from April 1^st^, 2020, to September 30^th^, 2021. The study protocol was authorized by the Siriraj Institution Review Board (approval no. Si-355/2020).

### Participant selection

We included all COVID-19 patients over 18 years of age with pneumonia who required oxygen therapy and were admitted to Siriraj Hospital. The COVID-19 diagnoses were confirmed by real-time reverse transcription–polymerase chain reaction. Patients who had “do not resuscitate” requests or declined endotracheal intubation were excluded from the study.

### Outcomes

The primary outcome of this study was in-hospital mortality. The secondary outcomes were ICU mortality and 28-day and 90-day mortality.

### Study procedure

We retrospectively reviewed the patients’ data, including demographic information, underlying diseases, duration of symptoms, hemodynamic parameters, Sequential Organ Failure Assessment (SOFA) score, and Acute Physiology and Chronic Health Evaluation II (APACHE II) score. We also collected data from laboratory investigations (arterial blood gas analysis, C-reactive protein, interleukin-6, complete blood count, blood chemistry, and liver function tests). Written informed consent was waived due to the retrospective study design, and approval was obtained from the Siriraj Institution Review Board (approval no. Si-355/2020). Data were accessed for research purposes from January 24, 2023, to April 15, 2023.

We recorded treatment interventions administered during hospitalization, such as antiviral drugs, corticosteroids, other anti-inflammatory agents (e.g., tocilizumab and baricitinib), hemoperfusion therapy, oxygen therapy, prone positioning, renal replacement therapy, and extracorporeal membrane oxygenation (ECMO) therapy provided by nurses, according to the physician’s order. The type and daily dose of corticosteroids given to each patient were recorded for the first 14 days and expressed as the dexamethasone-equivalent dose. The cumulative dexamethasone-equivalent dose during the initial 14 days was used to determine the intensity of corticosteroid treatment. We documented concurrent oxygen therapies administered during the same period. The table for calculating the dexamethasone-equivalent dose is shown in **S1 Table**. In addition, we reviewed potential complications associated with corticosteroid therapy, including hospital-acquired infections including hospital-acquired pneumonia, urinary tract infection, bacteremia, catheter-related bloodstream infection, skin and soft tissue infection, and gastrointestinal bleeding.

### Statistical analyses

Normality tests were conducted for qualitative data. Normally distributed data are expressed herein as means and standard deviations, whereas nonnormally distributed data are presented as medians and interquartile ranges (IQRs). Categorical data are reported as frequencies and percentages. To identify risk factors significantly correlated with mortality outcome, comparisons between survivors and nonsurvivors were made via the chi-square test, Fisher’s exact test, Student’s t test, and the Mann–Whitney U test when appropriate. Variables with p < 0.05 in the univariate analysis were included in a multivariate analysis via a multiple logistic regression model. The cumulative dose of dexamethasone received by each patient in the first 14 days was calculated and divided into quartiles (Q1-Q4). Mortality outcomes and complications were compared via the chi-square test. Kruskal‒Wallis tests were used to analyze other clinical outcomes. Data analyses were performed via IBM SPSS Statistics, version 29 (IBM Corp, Armonk, NY, USA). Two-sided p values < 0.05 were considered statistically significant.

## Results

### Baseline characteristics

**Fig 1:**
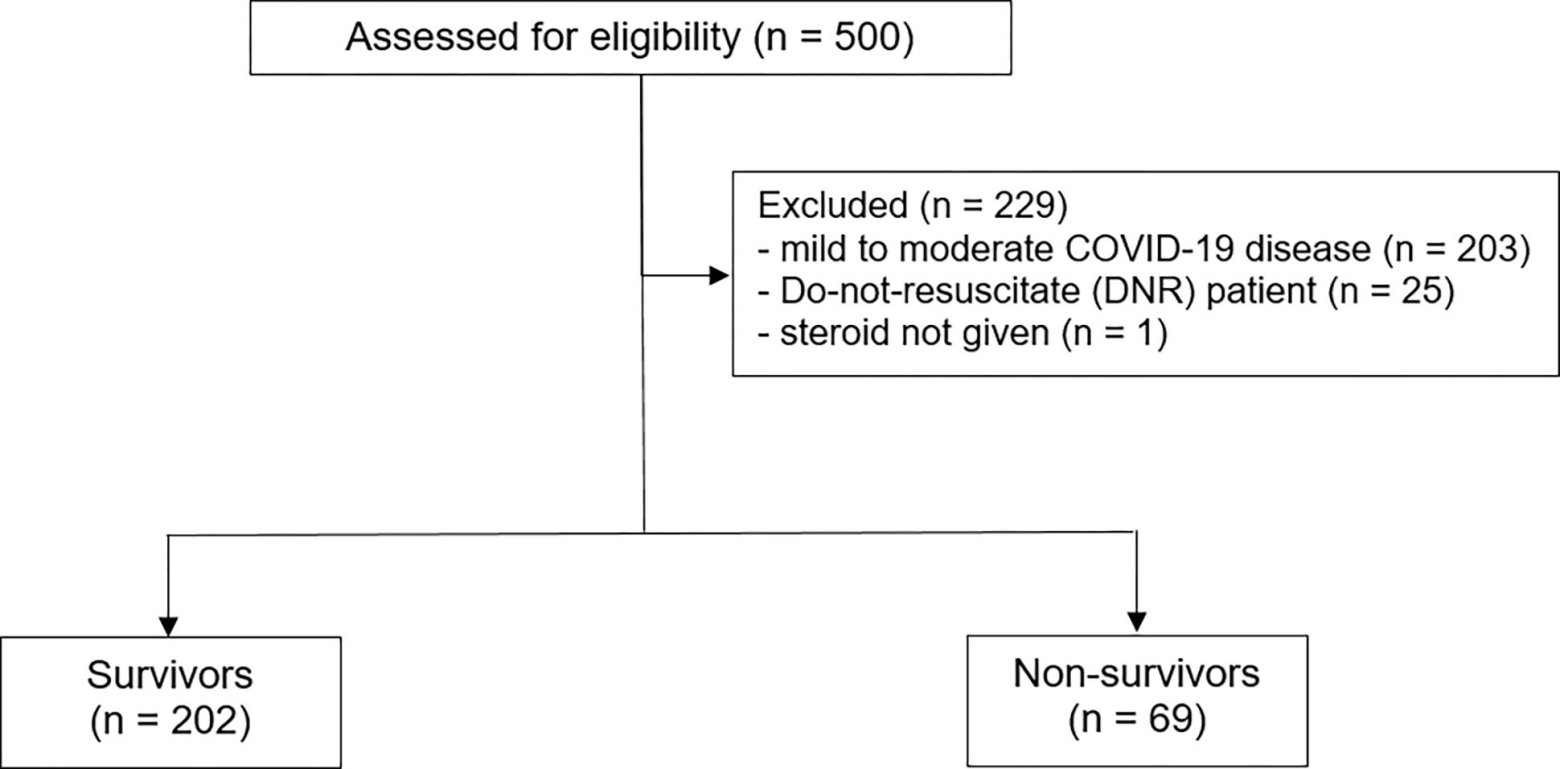
CONSORT flow chart.

Data were collected from 271 patients with COVID-19 pneumonia who required oxygen therapy at Siriraj Hospital between April 1st, 2020, and September 30th, 2021. Among these, 202 (74.5%) were in the survivor group, and 69 (25.5%) were in the nonsurvivor group (Fig 1). The characteristics of the study patients and treatment according to in-hospital mortality are summarized in **Table 1**. In the overall cohort, the median age of the patients was 62 years (IQR: 51, 69). Most patients were male, accounting for 66% of the study population. The baseline APACHE II and SOFA scores were 10 (IQR: 7, 13) and 3 (IQR: 2, 4), respectively. All patients had hypoxemia, with a median PaO_2_/FiO_2_ (PF) ratio of 151.4 (IQR: 104.8, 224.0). The median cumulative dexamethasone-equivalent dose administered in the first 14 days was 165 mg (IQR: 126, 203). In-hospital mortality occurred in 69 patients (25.5%). Survivors were younger, had a lower proportion of chronic kidney disease, and had lower severity scores (SOFA and APACHE II scores). In contrast, nonsurvivors had lower lymphocyte counts and PF ratios. Compared with survivors, nonsurvivors had a more significant dose of cumulative dexamethasone equivalence, received ECMO and mechanical ventilation more frequently, and had a longer duration of total ventilator days (**Table 1**).

**Table 1:** Baseline characteristics of the patients and treatment-related data according to in-hospital mortality.

variables	Survivors	Nonsurvivors	*p value*
	(n = 202)	(n = 69)	
Male, no. (%)	130 (64%)	50 (73%)	0.22
Age, years	57.82 ± 15.79	67.04 ± 12.28	< .001
Body mass index, kg/m^2^	27.45 ± 6.63	27.37 ± 6.04	0.93
Underlying diseases, no. (%)			
• Obesity (BMI > 30)	56 (27.7%)	22 (31.9%)	0.51
• Diabetes mellitus	90 (44.6%)	35 (50.7%)	0.38
• Hypertension	110 (54.4%)	49 (71%)	0.02
• COPD	5 (2.5%)	4 (5.8%)	0.18
• Coronary artery disease	19 (9.4%)	11 (15.9%)	0.14
• Chronic kidney disease	31 (15.3%)	22 (31.9%)	0.003
• Immunosuppression	10 (5%)	5 (7.4%)	0.46
APACHE II	9.5 ± 4.8	12.2 ± 4.9	0.001
SOFA	2.5 (2–4)	3 (2–5)	< 0.001
Laboratory investigations at baseline			
• C-reactive protein level, mg/L	73.2 ± 64	80.6 ± 79	0.44
• Lymphocyte count, cell/mm^3^	521.8 (303.7–960.2)	406.1 (287.3–660.5)	0.03
• Serum interleukin-6 level, pg/ml	20.8 (10.5–77.9)	21.0 (12.3–120.6)	0.1
• Serum procalcitonin level, ng/ml	0.2 (0.1–0.9)	0.2 (0.1–1.0)	0.10
• PaO2/FiO2 ratio	143.3 (112.6–178.6)	141 (99.3–185.4)	< .001
Treatment			
Cumulative dexamethasone-equivalent dose during D1-D14, mg	158 (119.9–197.25)	185 (131.7–222.0)	0.02
DOS before steroid treatment, day	5 (3–8)	4 (3–5.5)	0.43
Remdesivir, no. (%)	57 (28.2%)	28 (40.6%)	0.06
Tocilizumab, no. (%)	32 (15.8%)	16 (23.2%)	0.17
Baricitinib, no. (%)	7 (3.5%)	3 (4.3%)	0.74
Cytokine adsorptive therapy, no. (%)	29 (14.4%)	15 (21.7%)	0.15
ECMO, no. (%)	1 (0.5%)	6 (8.7%)	< 0.001
Ventilator requirement, no. (%)	78 (38.6%)	67 (97.1%)	< 0.001
Total ventilator days, days	5.5 (0–11.5)	8.5 (8–33.5)	< 0.001

Normally distributed variables are presented as the mean ± standard variation; nonnormally distributed variables are presented as the median (interquartile range).

Abbreviations: APACHE II, Acute Physiology and Chronic Health Evaluation II; BMI, body mass index; IQR, interquartile range; PaO2/FiO2 ratio: the ratio of arterial oxygen partial pressure to fractional inspired oxygen; SD: standard difference; SOFA: sequential organ failure assessment score; D1-D14, days since steroid therapy was initiated; DOS, day of symptom onset; ECMO, extracorporeal membrane oxygenation therapy; IQR, interquartile range.

### Outcomes

**Fig 2:**
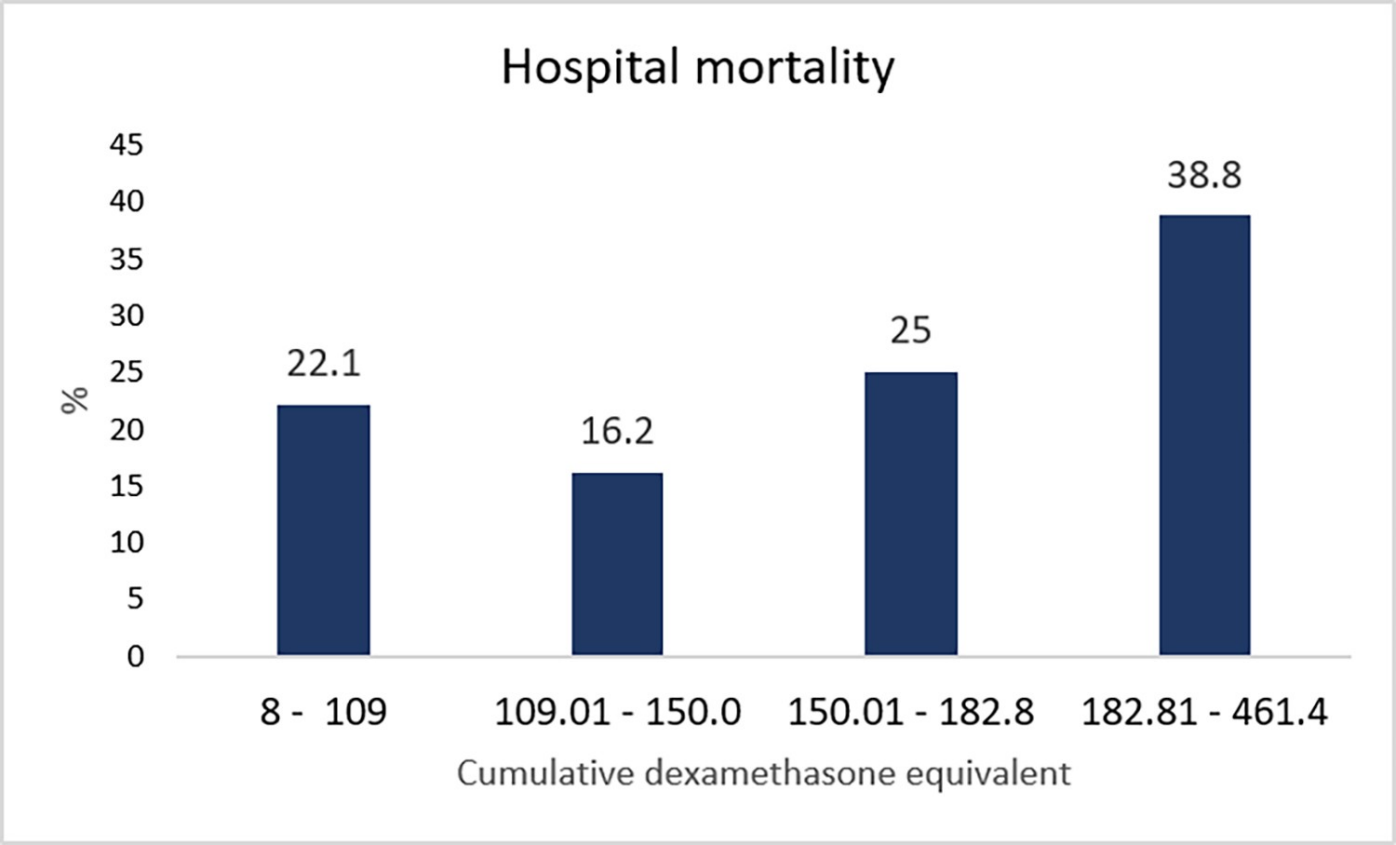
Hospital mortality based on the cumulative dose of dexamethasone. [a] statistically significant with *p* < 0.005.

In univariate analyses, risk factors, including age, chronic kidney disease, baseline SOFA score, lymphocyte count, baseline PF ratio, cumulative dexamethasone dose in the first 14 days, and ventilator requirement, were significantly associated with in-hospital mortality (**Table 1**). When these factors were included in the multivariable analysis, the risk factors that remained independently associated with in-hospital mortality were age, chronic kidney disease, baseline PF ratio, and ventilator requirement but not the cumulative dexamethasone dose (**S2 Table**). Although the cumulative dexamethasone equivalent dose was not statistically significant in the multivariable analysis, it remained a potentially modifiable risk factor during treatment. Therefore, patients were divided into quartiles on the basis of the cumulative dexamethasone dose in the first 14 days (8–126.00, 126.01–165.00, 165.01–203.00, and 203.01–481.40 mg) **(S3 Table)**. The second quartile group (126.01–165.00 mg/14 days) had the lowest in-hospital mortality rate at 16.2%, followed by the first, third, and fourth quartile groups at 22.1%, 25.0%, and 38.8%, respectively (**Fig 2**). The ICU mortality, 28-day mortality, and 90-day mortality rates showed similar patterns with statistical significance. Details of mortality and other clinical outcomes are presented in **Table 2**.

**Table 2:** Clinical outcomes stratified into quartiles according to the cumulative dexamethasone-equivalent dose in the first 14 days.

Clinical outcomes	Cumulative dexamethasone-equivalent dose (mg)				
	8–126.00	126.01–165.00	165.01–203.00	203.01–481.4	*p*—value
	[Q1]	[Q2]	[Q3]	[Q4]	
ICU mortality, no. (%)	10 (14.7%)	6 (8.8%)	12 (17.6%)	18 (26.9%)	0.01
In-hospital mortality, no. (%)	15 (22.1%)	11 (16.2%)	17 (25.0%)	26 (38.8%)	0.004
28-day mortality, no. (%)	14 (24.1%)	7 (11.5%)	10 (16.4%)	15 (23.4%)	0.29
90-day mortality, no. (%)	16 (29.1%)	12 (21.1%)	18 (32.1%)	26 (42.6%)	0.02
ICU LOS, days	8.5 (6–12)	10 (6.25–15.75)	11.50 (7–16.75)	16 (8–24)	< 0.001 ^a^^,^^b^
Hospital LOS, days	13 (9–17)	15 (11.25–24)	20 (14–31.75)	26 (17–40)	< 0.001 ^a^^,^^b^^,c^
Total ventilator days, days	7.5 (3.25–12.75)	10.5 (7–19.25)	9 (5.5–17.75)	20 (8–34)	0.003 ^a^
Ventilator-free days at day 28, days	28 (20.75–28)	27 (17.5–28)	23 (1.25–28)	2 (0–28)	< 0.001 ^a^^,^^b^^,^^c^^,^^d^
Hospital acquired infection, no. (%)	27 (39.7%)	38 (55.9%)	45 (66.2%)	52 (77.6%)	< 0.001
GI bleeding, no. (%)	7 (10.3%)	8 (11.8%)	7 (10.3%)	16 (23.9%)	0.06

Abbreviations: ICU, intensive care unit; IQR, interquartile range; LOS, length of stay; GI, gastrointestinal; Q, quartile

[a] Pairwise comparison revealed significant data with asymptotic significance (2-sided tests), with *p* < 0.05 between Q1 and Q4.

[b] Pairwise comparison revealed significant data with asymptotic significance (2-sided tests), with *p* < 0.05 between Q2 and Q4.

[c] Pairwise comparison revealed significant data with asymptotic significance (2-sided tests), with *p* < 0.05 between Q1 and Q3.

[d] Pairwise comparison revealed significant data with asymptotic significance (2-sided tests), with *p* < 0.05 between Q3 and QQ.

### Complications

The complications related to corticosteroids are shown in **Table 2**. Higher cumulative dexamethasone doses were significantly associated with an increased incidence of hospital-acquired infections (*p* < 0.001) and tended to increase the occurrence of gastrointestinal bleeding (p = 0.06).

## Discussion

In this retrospective cohort study of COVID-19 pneumonia patients, multivariate analysis revealed that the cumulative dexamethasone-equivalent dose was not a risk factor associated with increased in-hospital mortality. However, in the subgroup analysis, when we divided the cumulative dexamethasone-equivalent doses into quartiles, the second quartile (P25–P50; 126.01–165.00 mg/14 days) was significantly associated with the lowest mortality. Moreover, a greater cumulative dexamethasone-equivalent dose was associated with increased ICU LOS, hospital LOS and ventilator-free days at day 28.

The recommended dose and regimen of corticosteroids in COVID-19 pneumonia patients with hypoxemia remain inconclusive. Studies such as the COVID STEROID2 study and those conducted by Maskin LP et al. have shown trends favoring higher doses of dexamethasone, with benefits in days alive without life support and time required for cessation of mechanical ventilation [11, 12].

However, conflicting results have been reported, such as those of the randomized controlled trial by Toroghi et al., who found no clinical benefit of high-dose dexamethasone over conventional low-dose dexamethasone [13]. Similarly, the COVIDICUS trial did not demonstrate improved 60-day survival with a higher cumulative dexamethasone dose [14]. These discrepancies may be attributed to differences in corticosteroid intensity and disease severity across the various studies, as shown in **S4 Table**.

Our findings demonstrated the relationship between increased intensity of corticosteroids administered daccording to the hypoxemia severity and improved in-hospital mortality. This association was shown between the lowest intensity (dexamethasone equivalent < 126 mg) in patients with a median PF ratio of 290 (190–439) and a moderate intensity (126.01–165.00 mg) with a median PF ratio of 175 (129–268) (**Table 2 and Fig 2**). However, this association disappeared in patients with a greater degree of hypoxemia. Increasing the cumulative dexamethasone-equivalent dose beyond 165 mg was associated with increased in-hospital mortality.

This phenomenon could be explained by the fact that higher doses of steroids (> 165 mg) lead to an increased risk of hospital-acquired infections, as shown in **Table 2**. The incidence of secondary infections increased proportionally with an increasing cumulative dexamethasone dose, with statistical significance observed.

This “J-shaped” pattern aligns with the results of a retrospective study conducted by Maia et al. [15], which showed a strong correlation between the cumulative corticosteroid dose (expressed as a methylprednisolone equivalent) and the duration of mechanical ventilation. The shortest ventilation time was associated with a cumulative dose of 560 mg of methylprednisolone (which corresponds to 105 mg of dexamethasone). However, Maia et al. did not report other important clinical outcomes, particularly mortality.

The beneficial effects of higher corticosteroid doses on clinical outcomes have been demonstrated in studies with cumulative dexamethasone doses ranging from approximately 120 to 130 mg [11, 12]. These findings are consistent with our study, which showed that there was lower mortality when the cumulative dose of dexamethasone equivalence increased from low (< 126 mg) to moderate (126.01–165.00 mg) doses. Additionally, a study by Toroghi et al. revealed a trend of increasing 60-day mortality from 17% to 30% and 41% with cumulative dexamethasone doses of 80 mg, 160 mg, and 240 mg, respectively [13]. Similarly, in our investigation, cumulative doses of dexamethasone equivalent exceeding 165 mg were associated with increased in-hospital mortality.

The strength of our study lies in the consideration of both the cumulative dose and the timing of corticosteroid therapy. We followed clinical guidelines for personalized steroid modulation on the basis of patient severity and risk factors, adjusting the daily dose in response to parameters such as the degree of hypoxemia, chest radiography progression, and C-reactive protein levels [16]. Furthermore, the complications from corticosteroid therapy [17] were carefully monitored and recorded due to concerns about potential harm.

However, our study has several limitations. First, it was a retrospective, observational investigation with a limited number of patients and several unmeasured confounders. Thus, a larger trial is needed to confirm or refute these findings. Second, since the data were collected during the pandemic, therapeutic agents such as remdesivir, tocilizumab, and baricitinib were limited in availability, potentially influencing the clinical outcomes observed in this study. Third, we used the cumulative dexamethasone equivalent dose during the treatment period, which probably did not represent the daily dose of corticosteroids in COVID-19 patients with pneumonia in real-world practice. Fourth, our population varied in terms of age, comorbidities, days since symptom onset, and severity of disease, which may have resulted in heterogeneity in the clinical outcomes. Fifth, most of the patients in this study were recruited during the delta variant pandemic of SARS-CoV-2, which is not the current strain. Therefore, the results might not apply to the current variant. Sixth, our study reported hospital mortality as the primary outcome and mortality at different time points as secondary outcomes. We did not report the actual cause of death for each patient, which could have obscured a mechanism of death that might not be related to COVID-19. Seventh, we recruited only hypoxemic patients requiring corticosteroids, and thus, we could not compare the outcomes of these patients with those of patients not receiving corticosteroids.

## Conclusions

The intensity of corticosteroid therapy, measured as the cumulative dexamethasone-equivalent dose during the first 14 days, is not a risk factor for in-hospital mortality in hypoxemic COVID-19 patients. However, cumulative doses exceeding 165 mg are associated with higher in-hospital mortality and secondary hospital-acquired infection.

## Supporting information

S1 Checklist TREND statement checklist. (PDF)

S1 Table Dexamethasone equivalent dose. (DOCX)

S2 Table Univariable and multivariable analyses. (DOCX)

S3 Table Demographic data, treatment and clinical outcomes stratified into quartiles according to the cumulative dexamethasone equivalent dose. (DOCX)

S4 Table Previous study. (DOCX)

S1 Data (XLSX)

## References

[1] Triggle CR, Bansal D, Ding H, Islam MM, Farag EABA, Hadi HA, et al. A Comprehensive Review of Viral Characteristics, Transmission, Pathophysiology, Immune Response, and Management of SARS-CoV-2 and COVID-19 as a Basis for Controlling the Pandemic. Front Immunol. 2021 Feb 26;12:631139. doi: 10.3389/fimmu.2021.631139 33717166 PMC7952616

[2] Centers for Disease Control and Prevention. COVID-19 (coronavirus disease): people with certain medical conditions. 2020 Available at: http://www.cdc.gov/coronavirus/2019-ncov/need-extra-precautions/people-with-medical-conditions.html.Accessed March 28,2022.

[3] NIH. COVID-19 Treatment Guidelines. Clinical Management Summary. Last Updated: May 25, 2024. Available at: https://www.covid19treatmentguidelines.nih.gov/management/clinical-management-of-adults/clinical-management-of-adults-summary/. Accessed May 25, 2024.

[4] RECOVERY Collaborative Group, Horby P, Lim WS, Emberson JR, Mafham M, Bell JL, Linsell L et al. Dexamethasone in Hospitalized Patients with Covid-19. N Engl J Med. 2021 Feb 25;384(8):693–704. doi: 10.1056/NEJMoa2021436 32678530 PMC7383595

[5] Tomazini BM, Maia IS, Cavalcanti AB, Berwanger O, Rosa RG, Veiga VC, et al. Effect of Dexamethasone on Days Alive and Ventilator-Free in Patients With Moderate or Severe Acute Respiratory Distress Syndrome and COVID-19: The CoDEX Randomized Clinical Trial. JAMA. 2020 Oct 6;324(13):1307–1316. doi: 10.1001/jama.2020.17021 32876695 PMC7489411

[6] Sahu AK, Mathew R, Bhat R, Malhotra C, Nayer J, Aggarwal P, et al. Steroids use in non-oxygen requiring COVID-19 patients: a systematic review and meta-analysis. QJM. 2021 Nov 5;114(7):455–463. doi: 10.1093/qjmed/hcab212 34347106

[7] Dequin PF, Heming N, Meziani F, Plantefève G, Voiriot G, Badié J, et al. Effect of Hydrocortisone on 21-Day Mortality or Respiratory Support Among Critically Ill Patients With COVID-19: A Randomized Clinical Trial. JAMA. 2020 Oct 6;324(13):1298–1306. doi: 10.1001/jama.2020.16761 32876689 PMC7489432

[8] Angus DC, Derde L, Al-Beidh F, Annane D, Arabi Y, Beane A, et al. Effect of Hydrocortisone on Mortality and Organ Support in Patients With Severe COVID-19: The REMAP-CAP COVID-19 Corticosteroid Domain Randomized Clinical Trial. JAMA. 2020 Oct 6;324(13):1317–1329. doi: 10.1001/jama.2020.17022 32876697 PMC7489418

[9] Jeronimo CMP, Farias MEL, Val FFA, Sampaio VS, Alexandre MAA, Melo GC, et al. Methylprednisolone as Adjunctive Therapy for Patients Hospitalized With Coronavirus Disease 2019 (COVID-19; Metcovid): A Randomized, Double-blind, Phase IIb, Placebo-controlled Trial. Clin Infect Dis. 2021 May 4;72(9):e373–e381. doi: 10.1093/cid/ciaa1177 32785710 PMC7454320

[10] Edalatifard M, Akhtari M, Salehi M, Naderi Z, Jamshidi A, Mostafaei S, et al. Intravenous methylprednisolone pulse as a treatment for hospitalised severe COVID-19 patients: results from a randomised controlled clinical trial. Eur Respir J. 2020 Dec 24;56(6):2002808. doi: 10.1183/13993003.02808-2020 32943404 PMC7758541

[11] Durr KM, Hendin A, Perry JJ. Effect of 12 mg vs 6 mg of dexamethasone on the number of days alive without life support in adults with COVID-19 and severe hypoxemia: the COVID STEROID 2 randomized trial. CJEM. 2022 Apr;24(3):266–267. 35349129 10.1007/s43678-022-00293-1 PMC8962227

[12] Maskin LP, Bonelli I, Olarte GL, Palizas F Jr, Velo AE, Lurbet MF, et al. High- Versus Low-Dose Dexamethasone for the Treatment of COVID-19-Related Acute Respiratory Distress Syndrome: A Multicenter, Randomized Open-Label Clinical Trial. J Intensive Care Med. 2022 Apr;37(4):491–499. doi: 10.1177/08850666211066799 34898320 PMC8926919

[13] Toroghi N, Abbasian L, Nourian A, Davoudi-Monfared E, Khalili H, Hasannezhad M, et al. Comparing efficacy and safety of different doses of dexamethasone in the treatment of COVID-19: a three-arm randomized clinical trial. Pharmacol Rep. 2022 Feb;74(1):229–240. doi: 10.1007/s43440-021-00341-0 34837648 PMC8627167

[14] Bouaddma L, Mekontso-Dessap A, Burdet C, Merdji H, Poissy J, Dupuis C, et al. High-Dose Dexamethasone And Oxygen Support Strategies in Intensive Care Unit Patients With Severe COVID-19 Acute Hypoxemic Respiratory Failure: The COVIDICUS Randomized Clinical Trial. JAMA Intern Med. 2022 Sep 1;182(9):906–916. doi: 10.1001/jamainternmed.2022.2168 35788622 PMC9449796

[15] Maia R, Melo L, Mendes JJ, Freitas PT. Corticosteroids in COVID-19: A double-edged sword—a retrospective study. Med Intensiva (Engl Ed). 2022 Apr;46(4):229–231. doi: 10.1016/j.medine.2021.02.007 35461668 PMC9020163

[16] Thitayanapong A, Ratanarat R. Rational use of corticosteroid treatment in the early phase of severe COVID-19. Clinical Critical Care. 2023; 31. 10.54205/ccc.v31.259411

[17] Singer Mervyn, and Andrew R. Webb, ’ Miscellaneous drugs’, Oxford Handbook of Critical Care, 3 edn, Oxford Medical Handbooks (Oxford, 2009; online edn, Oxford Academic, 1 Aug. 2010), 10.1093/med/9780199235339.003.00020, accessed 24 Aug. 2023.

