# Supplementary material for: Associations between corticosteroid dosage and clinical outcomes in patients with hypoxemic COVID-19 pneumonia: A retrospective cohort study
Source: PLoS One. 2024 Sep 6;19(9):e0308069. doi: 10.1371/journal.pone.0308069 (PMC11379263; doi:10.1371/journal.pone.0308069)
Supplement: S1 Table — (DOCX) [file pone.0308069.s002.docx]

| **S1 Table. Dexamethasone equivalent dose** | |
| --- | --- |
| **Drug** | **Equivalent anti-inflammatory dose (mg)** |
| Dexamethasone | 0.75 |
| Methylprednisolone | 4 |
| Prednisolone | 5 |
| Hydrocortisone | 20 |
