# Supplementary material for: Associations between corticosteroid dosage and clinical outcomes in patients with hypoxemic COVID-19 pneumonia: A retrospective cohort study
Source: PLoS One. 2024 Sep 6;19(9):e0308069. doi: 10.1371/journal.pone.0308069 (PMC11379263; doi:10.1371/journal.pone.0308069)
Supplement: S2 Table — (DOCX) [file pone.0308069.s003.docx]

| **S2 Table. Univariable and Multivariable analyses** | | | |
| --- | --- | --- | --- |
| Variables | Univariate  OR (95% CI) | Multi-variate  OR (95% CI) | p-value |
| Sex, female, % | 0.67 (0.37 - 1.24) | - |  |
| Age, year | 1.05 (1.03 - 1.07) | 1.06 (1.03 - 1.11) | < 0.001 |
| BMI, kg/m^2^ | 1.00 (0.96 - 1.04) | - |  |
| Obesity, % | 1.22 (0.67 - 2.19) | - |  |
| Diabetes, % | 1.28 (0.74 - 2.22) | - |  |
| Hypertension, % | 2.05 (1.15 - 3.76) | 1.44 (0.60 - 3.50) | 0.42 |
| COPD, % | 2.46 (0.59 - 9.43) | - |  |
| CAD, % | 1.88 (0.80 - 4.01) | - |  |
| CKD, % | 2.58 (1.36 - 4.87) | 2.78 (0.92 - 9.01) | 0.08 |
| Immunosuppression, % | 1.50 (0.45 - 4.39) | - |  |
| APACHE II | 1.13 (1.05 - 1.21) | 1.03 (0.94 - 1.14) | 0.50 |
| SOFA Score | 1.38 (1.18 - 1.63) | - |  |
| C-reactive protein | 1.00 (1.00 - 1.01) | - |  |
| Lymphocyte count | 1.00 (1.00 - 1.00) | - |  |
| Interleukin-6 | 1.00 (1.00 - 1.01) | - |  |
| Procalcitonin | 1.02 (0.99 - 1.06) | - |  |
| PaO2/FiO2 | 1.00 (0.99 - 1.00) | - |  |
| Cumulative dexamethasone | 1.01 (1.00 - 1.01) | 1.00 (0.99 - 1.01) | 0.57 |
| Time to start steroid, days | 0.96 (0.88 - 1.05) | - |  |
| Remdesivir, % | 0.74 (0.98 - 3.07) | - |  |
| Tocilizumab, % | 1.60 (0.80 - 3.12) | - |  |
| Baricitinib, % | 1.27 (0.27 - 4.70) | - |  |
| Hemoperfusion, % | 1.66 (0.81 - 3.28) | - |  |
| ECMO, % | 19.14 (3.19 - 365.17) | 34.88 (3.86 - 833.35) | 0.005 |
| Ventilator used, % | 52.26 (16.07 – 330.06) | 22.28 (5.76 - 151.82) | < 0.001 |
