# Supplementary material for: Associations between corticosteroid dosage and clinical outcomes in patients with hypoxemic COVID-19 pneumonia: A retrospective cohort study
Source: PLoS One. 2024 Sep 6;19(9):e0308069. doi: 10.1371/journal.pone.0308069 (PMC11379263; doi:10.1371/journal.pone.0308069)
Supplement: S3 Table — (DOCX) [file pone.0308069.s004.docx]

| **S3 Table. Demographic data, treatment and clinical outcomes stratified into quartiles according by cumulative dexamethasone equivalent dose** | | | | | |
| --- | --- | --- | --- | --- | --- |
|  | **cumulative dexamethasone equivalent dose (mg)** | | | |  |
| **Variables** | **8–126.00** | **126.01–165.00** | **165.01–203.00** | **203.01–481.4** | **p-value** |
| Number of patients | 68 | 68 | 68 | 67 |  |
| Sex, female, % | 23 (33.8) | 16 (23.5) | 28 (41.2) | 24 (35.8) | 0.172 |
| Age, year | 62.00 [50.00, 72.00] | 62.00 [50.00, 69.00] | 61.50 [50.75, 72.00] | 64.00 [56.00, 72.50] | 0.621 |
| BMI, kg/m2 | 25.13 [22.11, 28.96] | 25.94 [23.00, 30.81] | 27.58 [23.15, 33.66] | 27.06 [23.85, 31.29] | 0.045 |
| *Co-morbidities* | | | | | |
| Obesity, % | 13 (19.1) | 19 (27.9) | 26 (38.2) | 20 (29.9) | 0.106 |
| Diabetes, % | 29 (42.6) | 30 (44.1) | 29 (42.6) | 37 (55.2) | 0.391 |
| Hypertension, % | 43 (63.2) | 37 (54.4) | 38 (55.9) | 41 (61.2) | 0.685 |
| COPD, % | 0 (0.0) | 5 (7.4) | 2 (2.9) | 2 (3.0) | 0.120 |
| CAD, % | 11 (16.2) | 10 (14.7) | 5 (7.4) | 4 (6.0) | 0.142 |
| CKD, % | 23 (33.8) | 11 (16.2) | 10 (14.7) | 9 (13.4) | 0.008 |
| Immunosuppression, % | 5 (7.4) | 5 (7.4) | 3 (4.4) | 2 (3.0) | 0.603 |
| APACHE II | 11.00 [7.50, 17.00] | 10.00 [5.75, 12.00] | 9.00 [7.00, 13.00] | 10.00 [8.00, 13.00] | 0.528 |
| SOFA score | 2.00 [1.00, 4.00] | 3.00 [2.00, 4.00] | 2.00 [2.00, 3.00] | 3.00 [2.00, 4.00] | 0.421 |
| *Baseline laboratory investigation* | | | | | |
| Lymphocyte count | 690.20 [420.21, 1114.55] | 639.09 [442.73, 967.86] | 560.24 [321.11, 939.64] | 503.02 [367.59, 848.39] | 0.095 |
| CRP | 60.88 [22.66, 112.61] | 61.00 [30.45, 101.28] | 62.45 [32.90, 100.28] | 45.79 [24.09, 74.48] | 0.285 |
| Interleukin-6 | 16.92 [3.81, 51.84] | 21.12 [12.38, 65.74] | 16.35 [11.80, 65.92] | 14.48 [9.86, 43.58] | 0.709 |
| Procalcitonin | 0.31 [0.12, 1.90] | 0.21 [0.09, 0.59] | 0.16 [0.08, 0.44] | 0.14 [0.08, 0.23] | 0.010 |
| PaO2/FiO2 | 290.00 [191.29, 435.50] | 175.17 [129.00, 267.81] | 143.08 [108.09, 227.62] | 129.54 [93.32, 182.14] | <0.001 |
| *Treatment* | | | | | |
| Dexamethasone, mg | 94.95 [71.50, 109.62] | 148.90 [139.75, 157.12] | 185.00 [177.60, 195.00] | 227.30 [214.65, 249.60] | <0.001 |
| Remdesivir, % | 16 (23.5) | 18 (26.5) | 28 (41.2) | 23 (34.3) | 0.111 |
| Tocilizumab, % | 6 (8.8) | 10 (14.7) | 18 (26.5) | 14 (20.9) | 0.043 |
| Baricitinib, % | 2 (2.9) | 2 (2.9) | 4 (5.9) | 2 (3.0) | 0.746 |
| Hemoperfusion, % | 8 (11.8) | 18 (26.5) | 7 (10.3) | 11 (16.4) | 0.046 |
| RRT, % | 13 (19.1) | 4 (5.9) | 4 (5.9) | 4 (6.0) | 0.014 |
| ECMO, % | 1 (1.5) | 0 (0.0) | 1 (1.5) | 5 (7.5) | 0.032 |
| Ventilator, % | 20 (29.4) | 34 (50.0) | 40 (58.8) | 51 (76.1) | <0.001 |
| Hemoperfusion, no | 0.00 [0.00, 0.00] | 0.00 [0.00, 1.00] | 0.00 [0.00, 0.00] | 0.00 [0.00, 0.00] | 0.100 |
| *Clinical Outcomes* |  |  |  |  |  |
| Hospital mortality, % | 15 (22.1) | 11 (16.2) | 17 (25.0) | 26 (38.8) | 0.020 |
| ICU mortality, % | 10 (14.7) | 6 (8.8) | 12 (17.6) | 18 (26.9) | 0.043 |
| 28-day morality, % | 14 (24.1) | 7 (11.5) | 10 (16.4) | 15 (23.4) | 0.226 |
| 90-day Mortality, % | 16 (29.1) | 12 (21.1) | 18 (32.1) | 26 (42.6) | 0.088 |
| ICU LOS, days | 8.50 [6.00, 12.00] | 10.00 [6.75, 15.25] | 11.50 [7.00, 16.25] | 16.00 [8.50, 24.00] | <0.001 |
| Hospital LOS, days | 13.00 [9.00, 17.00] | 15.00 [11.75, 24.00] | 20.00 [14.00, 31.25] | 26.00 [17.50, 39.00] | <0.001 |
| Ventilator, days | 0.00 [0.00, 2.00] | 1.00 [0.00, 10.25] | 4.50 [0.00, 9.25] | 12.00 [2.00, 27.00] | <0.001 |
| Ventilator free days, | 28.00 [22.25, 28.00] | 27.00 [18.50, 28.00] | 23.00 [3.75, 28.00] | 2.00 [0.00, 26.00] | <0.001 |
| *Complications* |  |  |  |  |  |
| GI bleeding, % | 7 (10.3) | 8 (11.8) | 7 (10.3) | 16 (23.9) | 0.064 |
| Hospital Infection % | 27 (39.7) | 38 (55.9) | 45 (66.2) | 52 (77.6) | <0.001 |
| *Abbreviation: APACHE II: Acute Physiological and Chronic Evaluation II; BMI: Body Mass Index; CAD: Coronary Artery Disease; CKD: Chronic Kidney Disease; COPD: Chronic Obstructive Pulmonary Disease; CRP: C-Reactive Protein; ECMO: Extracorporeal Membrane Oxygenation; GI: Gastrointestinal; ICU: Intensive Care unit; LOS: Length of Stay; RRT: Renal Replacement Therapy; SOFA: Sequential Organ Failure Assessment* | | | | | |
