# Supplementary material for: Associations between corticosteroid dosage and clinical outcomes in patients with hypoxemic COVID-19 pneumonia: A retrospective cohort study
Source: PLoS One. 2024 Sep 6;19(9):e0308069. doi: 10.1371/journal.pone.0308069 (PMC11379263; doi:10.1371/journal.pone.0308069)
Supplement: S4 Table — (DOCX) [file pone.0308069.s005.docx]

| **S4 Table. Previous study** | | | | |
| --- | --- | --- | --- | --- |
|  | COVID STEROID2  JAMA 2021  (N=982) [11] | Maskin LP et al.  J Intensive Care Med 2022  (N=100) [12] | Toroghi N et al.  Pharmacol Resp 2022  (N=133) [13] | The COVIDICUS RCT  JAMA 2022  (N=546) [14] |
| Protocol of iv dexamethasone | 12 mg iv od x 10 days   vs.  6 mg iv od x 10 days | 16 mg iv od × 5 days then  8 mg iv od × 5 days  vs.  6 mg iv od × 10 days | 8 mg iv tid x 10 days  vs.  8 mg iv bid x 10 days  vs.  8 mg iv od x 10 days | 6 mg × 10 days  vs.  20 mg × 5 days then  10 mg × 5 days |
| Cumulative dose of dexamethasone equivalent | 84 mg vs 42 mg | 120 mg vs 54 mg | 240 mg vs 160 mg vs 80 mg | 60 mg vs 90 mg |
| Inclusion criteria | Oxygen therapy > 10 LPM  Mechanical ventilation | COVID-19 related ARDS on MV < 72 h | Moderate to severe pneumonia  Require oxygen therapy | PaO2 < 70 mmHg  SpO2 < 90% (RA)  RR > 30/min  Need O_2_ > 6 LPM |
| MV support | 22% vs. 20% | 100% | 13% vs. 12.5 vs. 6.4% | 17.4% vs. 18.5% |
| PF ratio | 120 vs. 118* | 207 ± 73 vs 182 ± 55 | 168 ± 69 vs 195 ± 75 vs 210 ± 93 | NA |
| Clinical mortality | 28-day mortality  27% vs. 32%  (p = 0.10) | 28-day mortality  41% vs. 39%  (p > 0.999) | 60-day mortality  41% vs. 30% vs. 17%  (p = 0.06) | Overall mortality  27.3 % vs. 27%  HR 0.96 (0.69 to 1.33) |
