# Supplementary material for: Associations between corticosteroid dosage and clinical outcomes in patients with hypoxemic COVID-19 pneumonia: A retrospective cohort study
Source: PLoS One. 2024 Sep 6;19(9):e0308069. doi: 10.1371/journal.pone.0308069 (PMC11379263; doi:10.1371/journal.pone.0308069)
Supplement: Attachment — Submitted filename: Response to Reviewers July 8th, 2024.docx [file pone.0308069.s008.docx]

Professor Jiawen Deng

Editor-in-Chief,

PLOS ONE Journal

July 8^th^, 2024

Answer to review of: PONE-D-24-03848 ‘Effects of Corticosteroid Intensity on Clinical Outcomes in Hypoxic COVID-19 Patients

Professor Jiawen Deng

Thank you for your potential interest in publishing our work. We appreciate the valuable comments from the reviewers and believe that their suggestion improved our work. We have sent our manuscript to AJE academic English editing services for a grammar review and have corrected our manuscript according to their suggestions.

We have revised and added information to our manuscript in response to the reviewers’ comments, and below we provide responses to each issue raised.

All comments are restated below followed by our responses and description of the substance and location of any resulting changes made to the revised manuscript.

Yours sincerely

Napassorn Teeratakulpisarn

Thummaporn Naorungroj

Asso. Prof. Ranistha Ratanarat

Department of Intensive Care

Faculty of Medicine Siriraj Hospital Mahidol University

**Response to Reviewers**

**Reviewer #1:**

**- If the patient population factored into the decision to study dexamethasone quartiles, the title should reflected thst this is a study in severe, hypoxemic COVID-19 pneumonia**

**RESPONSE:** We thank the reviewer for this comment. We have changed the title to “Associations between corticosteroid dosage and clinical outcomes in patients with hypoxemic COVID-19 pneumonia: A retrospective cohort study”

**- having no patients receiving corticosteroid or no placebo group should be mentioned in the limitations section**

**RESPONSE:** We thank the reviewer for this thoughtful comment. We added the seventh limitation in P18, L 289-292 that “we recruited only hypoxemic patients requiring corticosteroids, and thus, we could not compare the outcomes of these patients with those of patients not receiving corticosteroids.”

**- while greatly improved, there are still many instances where the wording is awkward or confusing, while I do not have a specific institute/agency recommendation in mind, this manuscript would benefit from further English grammar review**

**RESPONSE:** We thank the reviewer for your comment. We have sent our manuscript to AJE academic English editing services for a grammar review and have corrected our manuscript according to their suggestions.
